# Remote Sensing the Phytoplankton Seasonal Succession of the Red Sea

**DOI:** 10.1371/journal.pone.0064909

**Published:** 2013-06-05

**Authors:** Dionysios E. Raitsos, Yaswant Pradhan, Robert J. W. Brewin, Georgiy Stenchikov, Ibrahim Hoteit

**Affiliations:** 1 Earth Science and Engineering (ErSE), King Abdullah University of Science and Technology (KAUST), Thuwal, Kingdom of Saudi Arabia; 2 Remote Sensing Group (RSG), Plymouth Marine Laboratory (PML), Plymouth, Devon, United Kingdom; 3 Met Office, FitzRoy Road, Exeter, United Kingdom; University of Vigo, Spain

## Abstract

The Red Sea holds one of the most diverse marine ecosystems, primarily due to coral reefs. However, knowledge on large-scale phytoplankton dynamics is limited. Analysis of a 10-year high resolution Chlorophyll-a (Chl-a) dataset, along with remotely-sensed sea surface temperature and wind, provided a detailed description of the spatiotemporal seasonal succession of phytoplankton biomass in the Red Sea. Based on MODIS (Moderate-resolution Imaging Spectroradiometer) data, four distinct Red Sea provinces and seasons are suggested, covering the major patterns of surface phytoplankton production. The Red Sea Chl-a depicts a distinct seasonality with maximum concentrations seen during the winter time (attributed to vertical mixing in the north and wind-induced horizontal intrusion of nutrient-rich water in the south), and minimum concentrations during the summer (associated with strong seasonal stratification). The initiation of the seasonal succession occurs in autumn and lasts until early spring. However, weekly Chl-a seasonal succession data revealed that during the month of June, consistent anti-cyclonic eddies transfer nutrients and/or Chl-a to the open waters of the central Red Sea. This phenomenon occurs during the stratified nutrient depleted season, and thus could provide an important source of nutrients to the open waters. Remotely-sensed synoptic observations highlight that Chl-a does not increase regularly from north to south as previously thought. The Northern part of the Central Red Sea province appears to be the most oligotrophic area (opposed to southern and northern domains). This is likely due to the absence of strong mixing, which is apparent at the northern end of the Red Sea, and low nutrient intrusion in comparison with the southern end. Although the Red Sea is considered an oligotrophic sea, sporadic blooms occur that reach mesotrophic levels. The water temperature and the prevailing winds control the nutrient concentrations within the euphotic zone and enable the horizontal transportation of nutrients.

## Introduction

**Figure 1:**
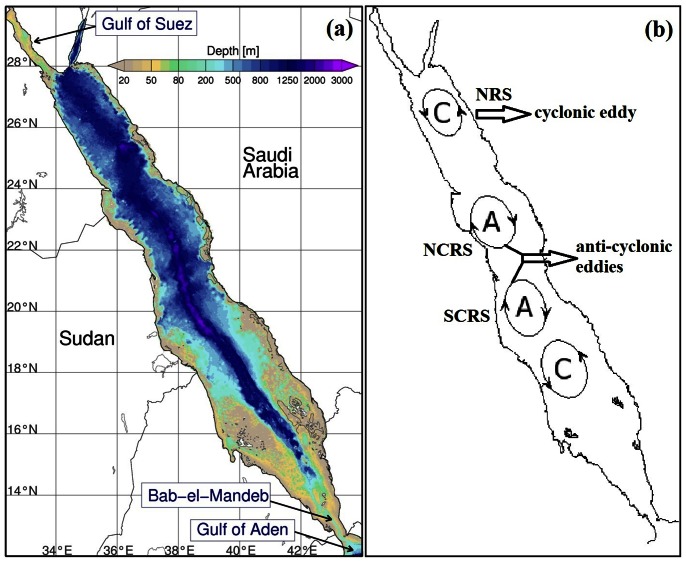
Bathymetry and general circulation of the Red Sea. **a)** Bathymetry of the Red Sea. At the southern part the Red Sea connects with the Gulf of Aden via the straits of Bab-el-Mandeb. **b)** Schematic representation of the general circulation of the Red Sea (figure copied and updated from Johns et al. [34]). The circular features represent the main cyclonic and anti-cyclonic eddies at the Red Sea.

The Red Sea Large Marine Ecosystem (LME) is an important economic and environmental asset [1]–[2]. This narrow oceanic basin (Fig. 1) is meridionally elongated (2250 km), lying between the African and the Asian continental shelves. A deep trench stretches from north to south along the axis of the entire sea reaching maximum depth of about 2300 m. At its northern end the Red Sea connects with the Mediterranean Sea through the Suez Canal, while the southern region exchanges its waters with the open ocean (Gulf of Aden and the Arabian Sea) via the strait of Bab-el-Mandeb [3]. The Red Sea LME provides habitats for a wide range of marine species some of which are endemic [4]. The Red sea is home to pristine coral reef ecosystems that have adapted to live in one of the most saline and warm seas in the world [1], [5]. This unique environment provides conditions which are likely to occur in other LMEs several decades from now, assuming predicted global warming and climate change scenarios materialise [6]. In addition to being a very warm environment, the Red Sea has been clustered as a fast warming LME [1]. There is evidence of an abrupt temperature increase since the mid-90 s, which persists till present [5]. Oceanic warming may have a direct and/or indirect impact on marine entities and ecosystems, and thus it is important to monitor the relatively unexplored and fragile Red Sea ecosystem [7].

The microscopic marine algae, phytoplankton, prosper in almost every aquatic environment. They play a fundamental role in the marine food web, biogeochemical cycling and climatic processes [8]–[9]. Able to grow in large quantities, their spatiotemporal variations influence biodiversity trends of other marine organisms [10]. Phytoplankton are an important food supply for coral reef ecosystems as they transfer energy to higher levels of the marine food web [2], [11], [12]. This in turn, through fisheries, provides a major source of protein to many of the poorest people in the world [13]. There is a significant positive relationship between remotely sensed chlorophyll-a (Chl-a) concentration and coral reef fish larval supply [14]. Despite the importance of Red Sea phytoplankton, and in contrast with knowledge regarding the physical oceanographic environment, scientific literature describing biological dynamics is limited [15], particularly at large scales.

The Red Sea is considered as an oligotrophic domain, deficient in several major nutrients including: nitrate, ammonium, phosphate, and silicate [15]. The entire stretch of the Red Sea lacks inputs from rivers or stream sources, meaning the required nutrients for photosynthetic activity have to come through water intrusion (from the Gulf of Aden), the sub-surface (below the nutricline), or via aerial deposition. Higher nutrient concentrations occur in the southern areas, gradually declining towards the north [16], and this general pattern is reflected in primary production [17]. The reservoirs of nutrients in the Red Sea are trapped below the stratified zone, primarily due to the persistent pycnocline. High stratification levels and the resulting homogeneity of the northern Red Sea deep waters ultimately prevents deep water renewal, and thus limits nutrient availability and biological productivity in the euphotic zone [15]. However, there is evidence of seasonal blooms taking place during spring months in the north-western region [15], which may be attributed to the nutrient supply through the convergence zone formed by the collision of two coastal currents (the Eastern and Western Boundary Currents) [18]. As Acker et al. [15] demonstrated (Figure 7 in [15]), the collision of these two currents (one heading northwards and the other southwards) fuels cold nutrient rich waters from deeper parts of the water column to rise to the surface.

Over the past two decades remote sensing measurements of ocean colour have provided unique information on surface marine productivity. The colour of the ocean is a good indicator of the primary photosynthetic pigment found in phytoplankton, chlorophyll-a [19]. Here, we assess the spatiotemporal distribution and abundance of surface chlorophyll in relation to the general circulation of the Red Sea using remotely sensed data.

## Methods and Data

### Satellite Remote Sensing Data

Temperature is an important parameter for phytoplankton growth, influencing it directly through physiological processes (metabolism, excretion, etc.) and indirectly through physical mechanisms such as stratification, nutrient availability and predation [20]. To identify the productive areas and define the seasonal succession in the Red Sea, high resolution (1 km) daily Chlorophyll-a (Chl-a) and Sea Surface Temperature (SST) data from the Moderate-resolution Imaging Spectroradiometer (MODIS on-board the Aqua platform) covering 2003–2011 period were obtained from NASA's Oceancolor website (http://oceancolor.gsfc.nasa.gov). Standard NASA algorithms were used for both Chl-a (OC3) and SST (sst4) estimates. MODIS SST [21] and Chl-a [22] R2009 data were produced by the Ocean Biology Processing Group at the Goddard Space Flight Center [23]. Both datasets were quality checked using standard L2 flags prior to analysis. The quality controlled daily swath records were then used to create gridded products (using a sample averaging method) at daily, weekly, monthly, and seasonal time scales, at ∼2 km horizontal resolution. Additionally, Chl-a and SST climatology maps were generated to delineate the semi-permanent features and to address the relationship between Chl-a and SST. The seasonal time windows, defined in this study, were determined from the weekly Chl-a images (Winter: mid December – mid March; Spring: mid March – mid May; Summer: mid May – Mid September; Autumn: mid September – mid December). The Red Sea was divided into four distinct meridional domains. The decision of the provinces’ latitudinal boundaries was based upon the spatiotemporal distribution and abundance of surface Chl-a, taking into account the weekly and monthly composite maps of the Red Sea. The four provinces, consecutively starting from the North to South, are the Northern-Red Sea (NRS), the North-Central Red Sea (NCRS), the South-Central Red Sea (SCRS), and the Southern Red Sea (SRS).

Besides temperature, wind plays a crucial role in determining the spatial and temporal variability of phytoplankton blooms (via surface advection and Ekman pumping). In this study, we reconstructed a mean seasonal wind dataset based on the QuikSCAT-v4 monthly mean wind fields covering the period from 2000 to 2009 [24]. The mean *u* and *v* components of the wind data are available at a 1/4 degree spatial resolution. QuikSCAT data were produced by Remote Sensing Systems and sponsored by the NASA Ocean Vector Winds Science Team. Data are available at www.remss.com/data/qscat.

### Cruise Data

Oceanographic data were collected from the R/V ‘‘Oceanus’’ during October of 2008 from the west coast of Saudi Arabia (near 21°N) as part of the King Abdullah University of Science and Technology (KAUST) – Woods Hole Oceanographic Institute (WHOI) research cruises expedition programme. Continuous fluorescence vertical profiles were collected down to 200 m depth at each of the 35 stations. The fluorescence instrument attached on the CTDs was a WET Labs ECO-FLNTUs (FLNTURTD-964) fluorometer and has been pre and post cruise calibrated to assure high quality Chl-a measurements. The fluorescence profile measurements were used to observe the Deep Chlorophyll Maximum (DCM).

### Interpretation of MODIS Chl-a in Shallow Waters

A large portion of the southern Red Sea is relatively shallow (<50 m) compared to the central and northern parts. It is worth mentioning that we have not used the MODIS 'TURBIDW' flag in the quality control process for the generation of the spatial maps, in order to obtain a complete picture of the Chl-a distribution. However, this flag was used in the spatially-averaged timeseries plots to avoid potential erroneously high values. Remotely sensed Chl-a data have known limitations especially in shallow optically complex Case II waters, where suspended sediments, particulate matter and/or dissolved organic matter do not covary in a predictable manner with Chl-a [25]. For instance, scattering by sediments in turbid waters and underwater reflectance from shallow reef areas may result in relatively high water-leaving radiance in the near-infrared (NIR) wavelengths, which could overestimate the correction term. Therefore, the Chl-a data used in the analysis may be influenced (generally resulting in an overestimation) by the factors mentioned above, especially in the coastal waters and/or very shallow waters of the Red Sea (Fig. 1A). A proper validation of this variable should involve a ground-truth comparison with several hundred *in situ* observations of approximately equal spatiotemporal distribution. Due to the lack of adequate *in situ* data, a satellite validation has not yet been possible for the Red Sea. However, large effort is being put towards data collection and several studies/cruises are being carried out, thus in the near future a sufficient amount of *in situ* samples will be collected, and in addition to the past data, will allow for ground-truth satellite verification. It has to be acknowledged that the southern Red Sea domain is the most susceptible to Chl-a retrieval issues. Therefore, this province is treated separately and the conclusions should be considered as tentative. However, not all the coastal high Chl-a values are necessarily erroneous, as the large coral reef complexes may be sources of either nutrients or chlorophyll-rich detritus that enhance phytoplankton production near the reefs [15]. Acker *et al*. [15] reported that the optical characteristics of the Red Sea water column fall within satellite missions standards. The scope of the current study is to assess the general phytoplankton seasonal spatiotemporal variability in the Red Sea, regardless of absolute concentrations. A comparison of monthly climatologies of chlorophyll in the Red Sea derived using the standard OC3 algorithm and the GSM semi-analytical algorithm [26], indicate that the two algorithms yield similar temporal patterns (NRS r = 0.97, *p<*0.05; NCRS r = 0.94, *p<*0.05; SCRS r = 0.75, *p<*0.05; SRC r = 0.74, *p<*0.05), suggesting conclusions drawn here regarding the Chl-a seasonal succession are insensitive to the choice of MODIS-Aqua Chl-a algorithm.

## Results and Discussion

### General Chlorophyll Patterns and Temporal Succession

**Figure 2:**
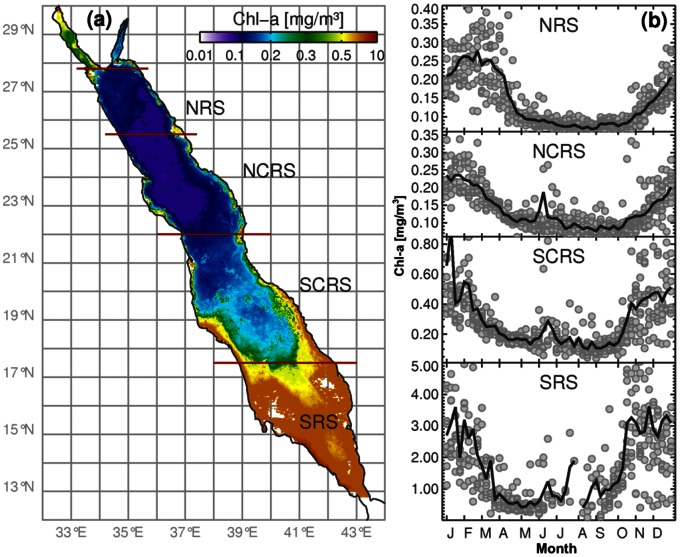
Phytoplankton biomass (Chlorophyll-a) in four provinces of the Red Sea. **a)** MODIS Chl-a (mg/m^3^) annual composite of the during (2003-present), at the Red Sea Chl-a. The four provinces consecutively starting from the North to South are the NRS, NCRS, SCRS, SRS. **b)** Weekly climatology of MODIS-Aqua Chl-a in the four provinces.

The characteristics of the spatial and temporal surface phytoplankton biomass are presented in this section. The annual Chl-a climatology (2003–2011) reveals several distinct patterns, that are related to the general circulation of the entire basin (Fig. 1B). The northern part is more oligotrophic than the south, splitting the Red Sea almost in half. The NCRS province exhibits the lowest Chl-a values and is less productive when compared with the NRS. In the southern Red Sea, the SRS appears to be the most productive province (Fig. 2A).

The seasonal succession of phytoplankton biomass for each region can be seen from the weekly mean time-series of Chl-a for the 2003–2011 period (Fig. 2B). Overall, the Red Sea Chl-a depicts a strong seasonal pattern with maximum concentrations seen during the winter time (attributed to vertical mixing in the north and intrusion of nutrient-rich waters from the Gulf of Aden in the south) and lowest during the summer (due to strong stratification and deeper pycnocline/nutricline). The initiation of the seasonal succession occurs in autumn and lasts until early spring. In every domain the initiation and the termination of the blooms are quite abrupt, indicating a distinct seasonality. However, with the exception of the SRS, the Chl-a concentrations do not exceed 1 mg/m^3^, indicating the oligotrophic nature of this region. Occasionally there are extreme blooms reaching several orders of magnitude higher than the weekly mean Chl-a values. Here, we define “bloom” as the area where higher Chl-a concentrations are observed than average, for example during the main growing season. Chl-a concentrations vary substantially along the Red Sea (from north to south), and thus due to their heterogenic nature, one cannot use a specific Chl-a concentration threshold to categorise a bloom in the entire Red Sea. For instance, in the NCRS province the peak of the Chl-a concentrations reach 0.4 mg/m^3^, whereas in the SRS reach 4 mg/m^3^. However, values greater than twice the average ambient chlorophyll concentrations (doubling) were used as an approximation to define blooms in oligotrophic water [27], [28], and a similar Chl-a threshold has been adapted in this work. The seasonal succession starts in mid October and ceases around the second week of March in the central and southern Red Sea, while in the NRS the blooms last longer by a month. Interestingly, although the Chl-a intensity reaches lowest levels during the summer, there is an evident peak in June that lasts approximately for a couple of weeks, apparent in all areas except in the NRS.

The NRS phytoplankton biomass exhibits the most distinct seasonal cycle, characterised by a sharp bloom initiation/termination, whereas the most irregular cycle can be seen in the SRS. As revealed by MODIS, the remotely-sensed Chl-a values during the peak periods starting from North to South reach on average 0.24, 0.2, 0.8 and 3 mg/m^3^ (for NRS, NCRS, SCRS, and SRS, respectively). Although the NRS depicts higher Chl-a values than NCRS during the winter, the reverse occurs during the summer. The phytoplankton phenology (timing of marine algae flowering) follows a relatively similar cycle over the entire Red Sea, where the bloom peak period is observed during mid-January, except in the NRS, where the seasonal succession peak occurs approximately four weeks later (end of February) and it remains higher for a longer period of time when compared to the rest of the provinces.

Two distinct characteristics of the SRS, which are not necessarily independent from each other, are the intense productivity and the shallow waters (Fig. 1A, Fig. 2). As discussed in section 2.3, however, the absolute values of satellite derived Chl-a should be treated with caution. Another feature, evident gradually from south to north, is that the Chl-a values are more variable (higher deviations) in the SRS compared with northern parts. Possibly lower precision in satellite Chl-a retrievals is not the only reason for these fluctuations. The southern areas are highly influenced by the intrusion of nutrient-rich waters from the Gulf of Aden and Arabian Sea [3]. Although the absolute SRS Chl-a values are probably biased, and appear to be several orders of magnitude higher (∼4 mg/m^3^) compared to the rest of the provinces (∼0.4 mg/m^3^), the overall seasonal trend is reproduced moderately well. Thus, regardless of uncertainty in satellite derived Chl-a concentrations in the SRS, it is important to analyse this area and report the seasonal patterns of this unique environment.

### Seasonal Patterns of Chlorophyll in Relation to the Physical Regime

**Figure 3:**
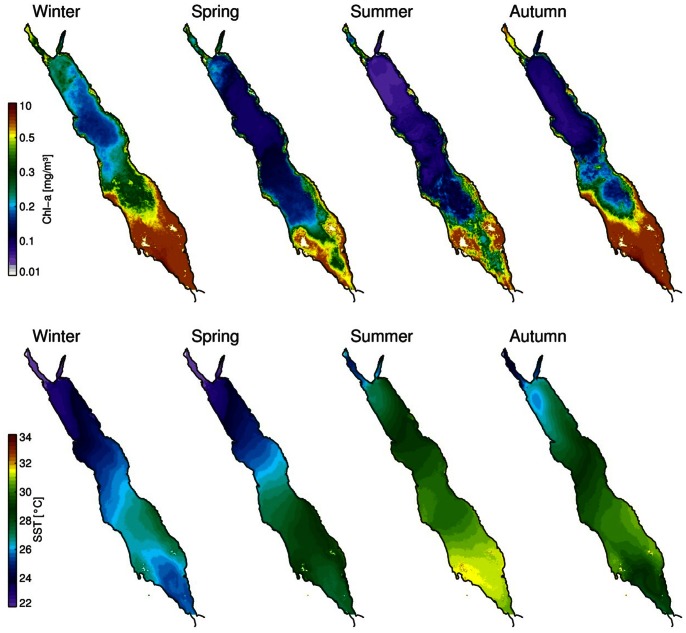
Red Sea seasonal climatologies of MODIS Chl-a (mg/m^3^) and SST (°C). The Local Area Coverage (LAC) dataset are presented for the period 2003–2011.

The seasonal succession of Chl-a in relation to SST and the prevailing wind over the Red Sea is reported in this section. The four seasonal climatologies of Chl-a and SST data were created from MODIS weekly composites (Fig. 3). The choice of four seasons was primarily to capture the four main Chl-a seasonal succession phases (autumn initiation, winter peak, spring termination and summer minima). The time window for the four distinct seasons is described earlier (see section 2.1). It is noticeable that each seasonal mean (climatology) is not derived from an equal number of weeks. However, a comprehensive analysis showed that the main alterations of Chl-a fall consistently within these time windows. The SST data can be interpreted as a proxy of deep vertical mixing (cold) or strong stratified (warm) waters.

The Chl-a concentrations in the NRS are higher during the winter and spring seasons, which co-occur with cold nutrient rich waters (deep mixing) (Fig. 3). As evident from the seasonal timeseries (Fig. 2B), the NRS summer minima is the lowest seen in space and time. The NRS SST is permanently cooler throughout the year compared to the rest of the Red Sea, indicating a permanent system (cold-core cyclonic pattern) that keeps replacing the surface waters with colder ones from deeper parts of the water column (Fig. 1B). As revealed by a three-dimensional Red Sea circulation model, this feature acts as a preconditioning mechanism to weaken the stratification [29]. From the Chl-a and SST images in Figure 3 it is apparent that the surface circulation in the NRS is cyclonic in nature, as reported in the past [2]. The seasonal succession timeseries plots (Fig. 2B) indicate that the initiation of the phytoplankton succession occurs at the beginning of autumn period, which is probably triggered by these cold and nutrient rich (through the convective mixing) waters (Fig. 3). This short delay in Chl-a response is probably due to the time needed for phytoplankton to utilise the nutrients before they grow. This delay is particularly evident from the weekly SST and Chl-a maps (not shown). At the NCRS the maximum Chl-a concentrations occur during the winter time but depict the lowest concentrations in the entire Red Sea during this period.

During the summer oligotrophic period, although the lowest Chl-a concentrations occur, a circulation feature seems to be responsible for the higher Chl-a values in the central Red Sea - a signal that is evident during June in Fig. 2B. Based on remotely-sensed data, anti-cyclonic mesoscale eddies seem to be responsible for the higher productivity (Fig. 1B). The NCRS anti-cyclonic eddy is apparent every June, lasting for approximately 2–3 weeks, and disappearing in July. These intense eddies occupy large areas of NCRS and SCRS, surrounded by coral reef structures from both sides of the Red Sea. It is thought that these mesoscale eddies play an important role in the regional ecosystem by transporting nutrients and/or Chl-a (through advection) to the oligotrophic zones during summer. The presence of such eddies (June 2001) have also been reported earlier [15]. The nutrient source may come from the adjacent coral reef ecosystems [15], and/or via aerial deposition (e.g., wind-blown dust that may carry micro-nutrients). Edward [30] reported that the southern part of the Red Sea is influenced by the spring and fall monsoon, as it affects the prevailing wind direction and thus dust transportation from the surrounding deserts. A compilation of all major dust reports (2002–2011) from NASA's rapid response system reveals that the maximum number of dust storms over the Red Sea have occurred during June-July (data not shown). However, further investigation is required to fully unravel the causes of this permanent feature and ascertain why it results in an increase in primary production.

**Figure 4:**
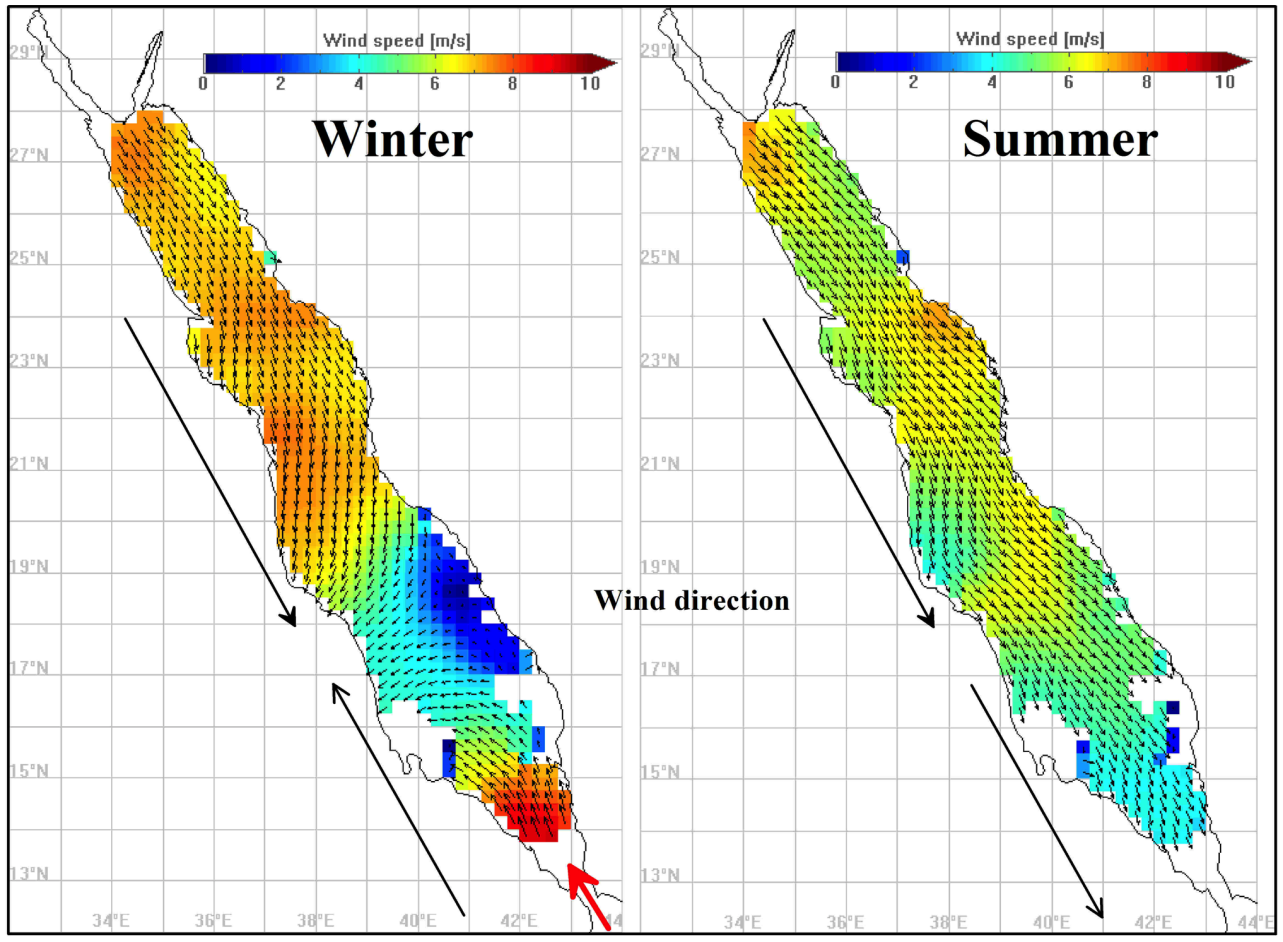
Red Sea seasonal climatologies of QuikSCAT wind speed (m/s), for the period 2000–2009. Winter climatology is based on averages from October to April and summer climatology from May to September). The black arrows highlight the different wind direction between the two seasonal climatologies, whereas the red arrow indicates the intrusion of water masses through the facilitation of intensive northwards winds.

In the southern Red Sea the SCRS is the second most productive area. It portrays higher Chl-a values in coastal waters and lower in the deeper central areas. Although the SCRS follows the typical Red Sea Chl-a seasonality, of higher winter and lower summer concentrations, during the warmer period high Chl-a values are apparent (in the deeper areas too). A strong anti-cyclonic eddy at approximately 19°N is probably responsible for this seasonal pattern, with lower productivity (warmer waters) at its core surrounded at its periphery by higher productivity (colder waters). This eddy is often seen with another anticyclonic system to its north along the western shore, mainly during the summer (Fig. 1B). In the SCRS the initiation of the blooming period occurs in autumn, a period that overlaps with the abrupt change in wind direction with north westerlies in summer and south easterlies in winter (Fig. 4). The north-south SST gradient (cooler in north) breaks down at the onset of autumn (Fig. 3), which overlaps with the change in wind direction, mainly in the SCRS and SRC. Clear evidence of cold water intrusion from the adjacent Gulf of Aden via Bab-el-Mandeb [30] is observed with a change in surface temperature in the SRS during autumn (Fig. 3). Despite uncertainty in the accuracy of MODIS Chl-a values in shallow waters, the SRS portrays the most productive part of the Red Sea (Fig. 2 and 3). Here, Chl-a peaks during autumn and winter and declines gradually from late spring to the end of summer. The summer time surface Chl-a minima in the SRS overlaps with the warmest SST in the region, which also coincides (as in the SCRS) with consistent north-westerly winds (Fig. 4). The prevailing winds in the northern half of the Red Sea do not exhibit significant seasonal variations (general direction remains southward during every season). However, a noticeable change in both wind speed and wind direction (northward) is observed during the autumn-winter period in the southern half of the Red Sea (Fig. 4). During this period, these strong south-easterly winds are likely the key drivers responsible for the intrusion of waters from the Gulf of Aden (Fig. 3). In addition, excessive evaporation and low precipitation in the Red Sea basin (about 2 m/y) forces a constant influx of Arabian Sea surface water through the strait of Bab-el-Mandeb in the south [2]. The synergy of the wind and evaporation facilitates an influx of relatively cold, low salinity, nutrient rich waters in the Red Sea, which makes the SRS a more productive region when compared with the rest of the provinces. The intrusion of waters is particularly apparent in the autumn-winter SST plots (Fig. 3), which reveal an anti-cyclonic circulation pattern (fully matured in winter) in the southern SRS with northward advection of cold surface waters along the western side of the SRS. The wind has a different influence in the NRS, where the winter time wind jets can cause significant evaporation and ocean heat loss which may potentially drive deep convection in that region [31]. The winter convective mixing plays a significant role in supplying nutrients to the euphotic layer of the water column. A cold-core eddy in the north-western NRS (Fig. 1B) in winter is likely to cause strong vertical mixing and result in elevated surface Chl-a concentrations (Fig. 3).

### Vertical Profiles of Chlorophyll-a

**Figure 5:**
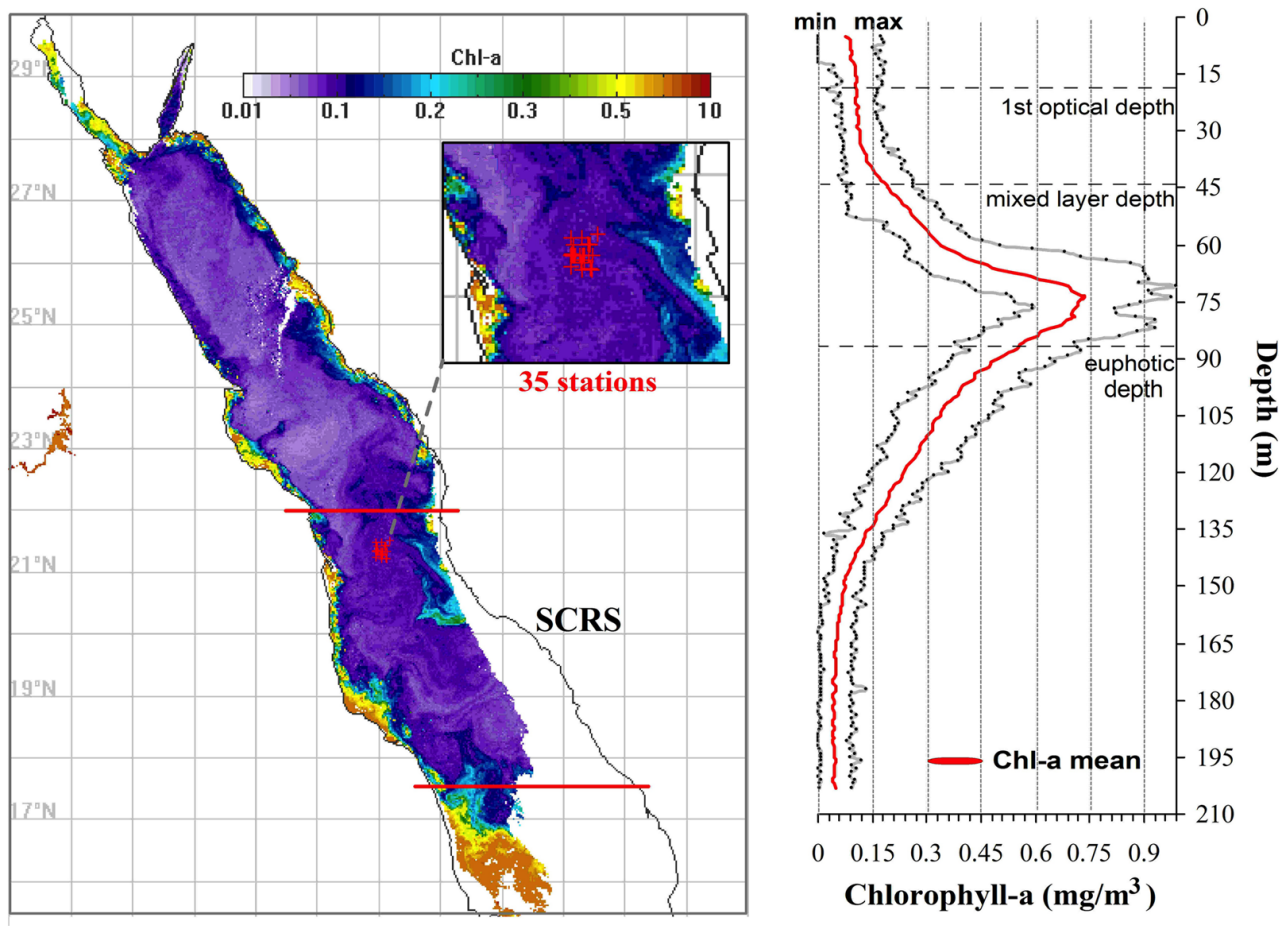
Vertical distribution of Chl-a profiles during October 2008 at the SRCS domain of the Red Sea. The average of 35 stations/profiles is depicted for each depth, along with the minimum and maximum profile range. The horizontal dashed-lines represent the 1^st^ optical depth, the mixed layer depth and the euphotic depth.

Satellites sensors can measure Chl-a concentrations in the top layer of the water column. The penetration depth of the observed signal depends mainly on light attenuation with depth (which in turn depends on the constituents present in the water column). We computed the average euphotic depth (1% light level) from the *in situ* chlorophyll concentrations for the 2008 cruise as 86.5 m (±9.3 m) and the 1^st^ optical depth as 18.8 m (±2.0 m) using the method of Morel et al. [32]. In open waters the chlorophyll maximum (or DCM) depth varies with geographic region and time of year. The DCM is a permanent feature in most tropical areas. It is likely that the satellite derived Chl-a measurements may not capture this information especially when the DCM is deeper than the optical depth. The DCM may indicate the maximum phytoplankton biomass in the water column, or it may indicate higher Chl-a concentrations per unit carbon from phytoplankton that dwell in a darker environment. Higher satellite-derived Chl-a concentrations may also reflect that the DCM is being brought to the surface by increases in the mixed layer depth (MLD) or changes in seasonal light, such that the depth integrated Chl-a does not change despite increases at the surface. However, a greater number of *in situ* measurements are needed to ascertain the causes of fluctuations in surface Chl-a. In general, surface phytoplankton biomass in stratified oligotrophic areas is limited primarily due to lack of nutrients. Under these conditions, the DCM is typically indicative of the nutricline. In this study, we have used *in situ* measurements of Chl-a profiles covering the central Red Sea area obtained from an oceanographic cruise during October 2008 (Fig. 5). Based on the overall average (35 stations) of *in situ* vertical profiles, a distinct DCM is apparent, with Chl-a concentrations reaching 0.7 mg/m^3^ (range: 0.6–1 mg/m^3^) at approximately 75 m depth. The MLD in this area was estimated at 44.4 m (±5.6 m). The MLD was computed as the depth in which the temperature changed by 0.5°C relative to the surface temperature based on Monterey and Levitus [33]. From the surface to 35 m depth, the mean Chl-a concentration during this period was 0.125 mg/m^3^ (range: 0.125–0.17 mg/m^3^). The base of the DCM falls within 35 m and 120 m. Column average (surface −30 m) *in situ* Chl-a is found to be of similar magnitude (∼0.1 mg/m^3^) as compared with the MODIS Chl-a values. This analysis shows that MODIS Chl-a is not representative of the DCM in the SCRS. However, more *in situ* validation is required to get a comprehensive understanding of the performance of MODIS Chl-a in the Red Sea.

### Conclusions

The analysis presented in this paper provides a detailed description of the spatiotemporal seasonal succession of surface phytoplankton biomass in the Red Sea based on satellite remote sensing data. *In situ* measurements of Chl-a in the SCRS revealed a marked DCM at 75 m, which was not captured by MODIS Chl-a estimates since the estimated 1^st^ optical depth (18.8 m) was shallower than the DCM depth. Supporting physical data, such as temperature (proxy of nutrient availability) and wind (proxy of circulation/water masses movement), were used to assess the bio-physical characteristics of the region. Based upon high resolution datasets, four distinct Red Sea provinces and seasons are suggested, which cover the main spatiotemporal patterns of surface phytoplankton production. It is evident that Chl-a concentration does not increase regularly from north to south, as had been thought before [17]. The Red Sea Chl-a depicts a strong seasonal pattern with maximum concentrations seen during the winter time (attributed to vertical mixing in the north and wind-induced horizontal intrusion of nutrient-rich water in the south), and minimum concentrations during the summer (due to the strong seasonal stratification). The initiation of the seasonal succession occurs in autumn and lasts until early spring. In every domain the initiation and the termination of the blooms are quite abrupt, indicating a distinct seasonality. The NCRS appeared to be the most oligotrophic area, probably because of the absence of a deep mixing mechanism as seen in the NRS, and due to lower nutrient availability from water intrusion (via the Gulf of Aden). The weekly Chl-a succession, as well as the seasonal maps contribute information intended to help better understand the patchy nature of the phytoplankton biomass distribution, and their proximity from the coral reefs. The results presented here further provide essential information for validating ecosystem models that are being developed for the Red Sea.

Although the Red Sea is considered an oligotrophic sea, we have observed sporadic blooms in the open oceanic parts and/or the coastal reef areas that reach mesotrophic levels. In oligotrophic waters, such as the Red Sea, the limiting factor for phytoplankton abundance is the availability of nutrients. In the absence of any riverine inputs, nutrients become available either through vertical mixing, intrusion of water masses (via Bab-el-Mandeb), aerial deposition, and/or possibly from coral reefs themselves. Water temperature and wind plays an important role in the productivity of the Red Sea. They act synergistically to control the nutrient level at the euphotic zone (through convection), and facilitate the horizontal transportation of nutrients (through advection). Our analysis confirm previous modelled studies, and show clearly the importance of the wind, as a key variable for the intrusion and the transfer (advection) of colder nutrient-rich waters from Gulf of Aden (via Bab-el-Mandeb) into the Red Sea. During autumn until early spring (Oct-Apr) the wind direction is reversed over the southern half of the Red Sea, and enables the northward advection of nutrients. This phenomenon coincides with the initiation and duration of the main phytoplankton growing period of the Red Sea.

The relationships between the Red Sea coral reefs and the primary producers are still unexplored and tentative. However, there is probably a two way mutual feedback mechanism between them. As Acker *et al.* [15] argued, and as we have seen from our analysis, the coral reefs seemed to fuel nutrients and/or Chl-a to open oceanic regions, which in turn shape phytoplankton blooms and support the early larval stage of reef dwellers [14]. In this study, satellite derived Chl-a data reveal that seasonal anti-cyclonic eddies (during June), in the central parts of both northern and southern provinces, transfer nutrients and/or chlorophyll to the open waters of the central Red Sea. The origin of the nutrients in those coral reef ecosystems is still tentative and requires further investigation. This phenomenon occurs during the stratified nutrient depleted season, and thus could provide an important source of nutrients to the open waters.
